# Physiological, Anthropometric, and Motor Characteristics of Elite Chinese Youth Athletes From Six Different Sports

**DOI:** 10.3389/fphys.2019.00405

**Published:** 2019-04-12

**Authors:** Kewei Zhao, Andreas Hohmann, Yu Chang, Bei Zhang, Johan Pion, Binghong Gao

**Affiliations:** ^1^School of Kinesiology, Shanghai University of Sport, Shanghai, China; ^2^Department of Sport Science, University of Bayreuth, Bayreuth, Germany; ^3^Shanghai Sports School, Shanghai, China; ^4^Hogeschool Arnhem and Nijmegen, Faculty of Education, Institute of Sport and Exercise Studies, HAN University of Applied Sciences, Nijmegen, Netherlands; ^5^School of Physical Education and Sport Training, Shanghai University of Sport, Shanghai, China

**Keywords:** talent, youth sport, discriminant analysis, neural network, test validity

## Abstract

Several talent selection programs in elite sport schools are based on motor diagnostics for the purpose of recommending or transferring promising talents to general groups of sports; game sports, combat sports or endurance sports, and to more concrete sports such as gymnastics, skiing, or tennis. However, the predictive value of such testing is unclear. This study evaluated the concurrent validity of physiological performance prerequisites, body dimensions, as well as specific motor performances. The sample consisted of *N* = 97 youth athletes from all ninth grade classes of a Shanghai Elite Sport school belonging to six different sports including basketball (*n* = 7), fencing (*n* = 23), judo (*n* = 20), swimming (*n* = 10), table tennis (*n* = 15), and volleyball (*n* = 22). The performance diagnosis took place between September 2016 and March 2017, and comprised five physiological measurements of the heart rate at rest, vital capacity, systolic and diastolic blood pressure, and hemoglobin concentration in the blood, eighteen anthropometric parameters, and two motor tests on back strength and complex reaction speed. The aim of the study was to investigate whether U15 age group athletes participating in six different sports already at this age show a sport specific anthropometric, motor performance, and physiological profile which is in line with the specific requirements of each of the sports. A discriminant analysis and a Neural Network (Multilayer Perceptron) were used to test whether it is possible to discriminate between athletes of the six sports and to assign each individual of the Under-15 athletes to his own sport on the basis of a unique profile of the morphological, motor, and physiological prerequisites. All diagnostic methods exhibited medium to high validity to discriminate between the six different sports. The relevance of the eighteen body dimensions, five physiological measures, and two motor tests for talent identification was confirmed.

## Introduction

Participation in elite sport training at youth age is associated with the selection of athletes with specific prerequisites and the development of the specific anthropometric, motor and physiological characteristics of a particular sport (Pion et al., 2015). For example, judo athletes at age U13 exhibit a higher sideward jumping ability compared to Karate and Taekwondo athletes (Pion et al., 2014). In fencing, Krishnan et al. (2017) could not find a superior broad jump ability in U21 fencers compared to weightlifters and wrestlers. In swimming, Bencke et al. (2002) found that male gymnasts showed better jumping abilities compared to swimmers, handball, and tennis players. Opstoel et al. (2015) also found in their study that U12 year old swimmers did not differ from ballsports players, dancers, gymnasts, martial arts and other sports participants in twelve different motor tests, although they were slowest in ball dribbling. In table tennis, Maehner (2018) found U15 players to be superior to soccer players in sideward jumping and push-ups. In volleyball, talented youth players are characterized by a higher stature as well as by a better jumping ability (Rikberg and Raudsepp, 2011). In basketball, at least female players exhibit a lower level of static and dynamic balance compared to gymnasts and soccer players, respectively (Bressel et al., 2007). On the basis of such findings, the discriminating sports specific characteristics can be recommended for talent identification purposes. But, as all studies mentioned above were executed with caucasian athletes from Europe, there is a lack of knowledge in regard to the make-up of Chinese youth athletes. To our knowledge this study is the first one comparing Chinese elite youth athletes from different sports in regard to sports specific characteristics. Thus, the purpose of this study was to investigate whether U15 age group athletes participating in six different sports under the condition of two daily training sessions in a Chinese elite sports school already at the U15 age show a sport specific anthropometric, motor performance, and physiological profile which is in line with the specific requirements of each of the particular sports, and which could serve as scientific knowledge background for sports specific talent identification purposes.

In long-term talent development programs, talent identification procedures include morphological measures and motor tests as well as physiological data. According to Pion et al. (2015), talent identification is related to homogeneous samples and aims at pinpointing the most promising young athletes to engage in long-term, elite sport training. Following this idea, several talent identification programs in elite sport schools have implemented morphological, motor, and physiological diagnostics (e. g., Hoare, 1995; Fuchslocher et al., 2011; Douglas, 2014; Kinugasa, 2014; Pion, 2015; etc.) to select or transfer young athletes into certain sport groups, such as combat sports, game sports, or endurance sports (Pion et al., 2014), and even more concrete to specific sports, like e.g., alpine skiing (Mueller et al., 2015). Talents in particular sport disciplines exhibit a specific make-up of natural abilities (nature) and well-developed performance prerequisites (nurture) (Pion et al., 2015). Therefore, the predictive validity of such talent characteristics is paramount when identifying promising youth athletes. Although some academics warn against talent identification procedures that are conducted too early (Meylan et al., 2010), these procedures targeted at juveniles are worthwhile for the purpose of helping sport federations maintain focus on their resources regarding the most talented young athletes (Unnithan et al., 2012; Höner and Votteler, 2016).

Many sports are based on a complex, multi-dimensional performance profile (Buekers et al., 2015). Thus, the talent selection should be focused on a multifaceted variety of general physical, physiological, psychomotor, and psychological performance diagnostics (Williams and Franks, 1998; Williams and Reilly, 2000). In general, there is a lack of research investigating the discriminative value of different performance prerequisites over a range of different sport disciplines. Nevertheless, there were promising attempts to discriminate various sports by means of their profile of sports specific performance prerequisites. So, Leone et al. (2002) could distinguish 88% of athletes from four different sports (figure skating, swimming, tennis, and volleyball) by means of a discriminant analysis including anthropometric and motor characteristics. Opstoel et al. (2015) reported a correct classification of 85.2% of high active U12 athletes into their own sport (ball sports, dance, gymnastics, martial arts, raquet sports, and swimming. Also, Pion et al. (2015) could assign 96.4% of 141 adolescent Flemish athletes into nine different sports. Even more promising were the findings of Pion et al. (2014) in elite male U18 athletes, as the investigators found a 100% correct classification within the more interrelated martial arts disciplines judo, karate, and taekwondo. In contrast to the aforementioned studies, the discriminant analysis is less accurate, when a hold-out of one case (*n* = 1) is used which has to be classified on the basis of the discriminant functions obtained from all other cases (n-1). Using this cross-validation strategy in a discriminant analysis with 56 12–16 years old youth athletes from six different sports (water polo, volleyball, soccer, cross-country skiing, running sprint, and alpine skiing), Hohmann et al. (2015) reported a correct assignment of 76.8%. Using alternatively the neural network method multilayer perceptron (MLP) the authors reported a lower classification rate of 69.6%.

Thus, reliable and valid information regarding the potential of talented athletes in certain sports on the basis of morphological parameters, motor abilities and skills, and physiological diagnostics is a valuable tool in talent development programs for clubs and sport federations. The main reasons for these scientific uncertainties in talent orientation arise from the often-undifferentiated mixture of general as well as sport-specific tests in talent identification campaigns; the unsystematic timing of cross-sectional diagnostics at single points in time during the long-term athletic development process also contributes to the aforementioned uncertainty. Thus, it is not surprising that the great variety of study design parameters have led to inconsistent research results, providing an inconsistent picture with regard to the discriminative validity of talent features addressing general and sport-specific performance prerequisites. Therefore, for talent orientation, there is a need for a multifaceted test battery that allows the ability to distinguish between the specific skills/physical attributes necessary for various sports.

Although the prediction of long-term success is still debatable, the talent orientation method of recommending suitable sports to children in accordance with their individual talent make-up seems feasible (Pion et al., 2015). Thus, the general aim of this study is to discriminate elite adolescent male athletes from a Shanghai Elite Sport school from seven different sports by means of morphological, motor, and physiological tests. It was hypothesized that a generic test battery consisting of 25 diagnostic tests has enough discriminative validity to assign athletes to their own sport on the basis of their individual profile of test scores.

## Materials and Methods

A sample of *N* = 97 Under-15 and Under-16 youth athletes from six different sports (age: *M* = 178.2 mon; *SD* = 6.9; *Min* = 168 mon; *Max* = 191 mon) attending the ninth grade classes of the Shanghai sports school took part in this study (see Supplementary Material). All athletes take part in 1–2 daily training sessions which amount to more than 20 h total training time per week (*M* = 20.8 h/w).

Due to the character of the Shanghai sport school as an institution that promotes peak performance athletes only, the athletes of the ten incorporated sports sections of the school had to be selected according to age and training history, so that the participants matching these criteria represent a resulting sample out of six sports only. As the track and field group consisted of athletes from six different disciplines (pole vault, long jump, high jump, hurdle sprint, running sprint, decathlon) it had to be cut out from the study. Three other disciplines (modern pentathlon, baseball, and badminton) did not comprise enough male athletes in the interesting U15 and U16 age group. The participants were recruited according to the ethical standards of the Shanghai University of Sports (SUS). Ethics approval and parental written informed consent was obtained from the participants of this study in accordance with the declaration of Helsinki. All athletes’ parents were informed about this study protocol, which was outlined in an information letter. No data collection took place without parents’ consent. All athletes were performing at a high level in their respective sport, representing China and/or the Shanghai province in international competitions.

### Measurements

The participants completed five physiological, eighteen morphological, and two motor tests that were administered by expert sport school staff members. All tests were conducted on the same day in both the gym and sport science laboratory on campus. The testing started at 10 a.m., and all athletes refrained from strenuous exercise one day prior to the test session.

#### Morphological Characteristics

Body height (BH) and sitting height (SH) to the nearest 0.1 cm (Height Tester, Donghuateng Sports Apparatus Ltd, Beijing, China), arm span (AS), arm length (AL), leg length (LL), lower leg length (LLL), shoulder width (SW), crista width (CW) to the nearest 0.1 cm (Martin Ruler, Donghuateng Sports Apparatus Ltd, Beijing, China), chest girth (CHG), calf girth (CAG), waist girth (WG), thigh circumference (TC), ankle circumference (AC) to the nearest 0.1 cm (Circumference ruler, Donghuateng Sports Apparatus Ltd, Beijing, China), Achilles tendon length (ATL) to the nearest 0.1 cm (Martin Ruler, Donghuateng Sports Apparatus Ltd, Beijing, China), subscapular angle (SA) to the nearest 1.0° (Protractor, Donghuateng Sports Apparatus Ltd, Beijing, China), abdomen skinfold thickness (AST), upper arm skinfold thickness (UAST) to the nearest 0.1 cm (Harpenden skinfold caliper, British Indicators, United Kingdom), and body weight to the nearest 0.1 kg (calibrated Seca Alpha 770) were measured according to standardized test prescriptions (Hawes and Martin, 2001; Stewart et al., 2011).

#### Motor Characteristics

Maximal dynamic back strength (measured by power dead lift) and simple reaction time (ms; PsyTech Sports; Xinyi Electronic Technology Company, Shanghai, China) were tested by expert staff members from the elite sport school.

In basketball (Chaouachi et al., 2009), as well as in volleyball (Bunn et al., 2017) maximal dynamic back strength turned out to be a relevant predictor of sport performance. Also, in judo it was shown by Drid et al. (2015) that elite athletes exhibit a higher maximum dynamic strength in deadlift and squat testing than their subelite counterparts. In fencing (Turner et al., 2014) as well as in crawl sprint swimming (Morouço et al., 2011), the power of the squat movement is a relevant predictor for the lunge speed, and the swimming power, respectively. Although there was no report on the validity of back strength testing in table tennis, the high reliability of the dead lift test (ICC = 0.99; Comfort and McMahon, 2015) allowed for the use of this measurement in all six sports of this study.

Before the dynamic back strength test, subjects performed a warm-up consisting of cycling and dynamic stretching. During the test the standardized procedures for the one repetition maximum (1RM) deadlift was followed (Hoffman, 2006). A low-intensity set of 5–10 repetitions was performed using 40–60% of the perceived 1RM. After a 1-minute rest, subjects performed a set of 2–3 repetitions at 60–80% of the perceived 1RM. Subsequently, subjects performed 3–5 maximal trials, followed by an assessment of 1RM deadlift strength.

Computerized measurements of simple reaction times show a sufficient reliability (ICC = 0.51; Eckner et al., 2011). Although it is known that game sports athletes show shorter simple reaction times than non-athletes, there is only few evidence for the validity of a simple reaction time assessment to distinguish between different sports (Badau et al., 2018). In this study, it was assumed that at least in the games sports (basketball, volleyball, and table tennis) and in the combat sports (fencing and judo) participants might exhibit different levels of performance in the computer-based test of the single-choice reaction time, especially when compared to swimming.

In the simple reaction time assessment the test device was prepared to measure the time of a simple response to light stimulation. The subject sat in front of the test instrument, placed his right index finger on the button, and pressed the button when the red light was on. The measurement included 20 repetitions, and the average value was calculated and used for all further data analysis.

#### Physiological Characteristics

Resting heart rate (bpm; Polar H10 Heart rate sensor, Polar Electro Inc., Finland), vital capacity (ml; High precision digital electronic spirometer, Donghuateng Sports Apparatus Ltd, Beijing, China), hemoglobin mass (mg; HemoCue Hb 201; HemoCue AB, Angelholm, Sweden), and blood pressure (mmHg; HEM-1000, Blood Pressure Monitor, Omron Health Care Inc., Japan) were diagnosed by medical personnel of the Shanghai University of Sport.

The long-term training effect of a reduced heart rate at rest in elite endurance sports is well known (Wilmore and Costill, 1994), wherereas up to now the adaptation of blood pressure parameters on sports performance was investigated primarily in strength sports, e.g., weightlifting (Dhamu et al. (2012). Generally, it is assumed that the training-induced decrease of the systolic blood pressure is more pronounced in weight training than in endurance sports (Hagberg, 1990).

Vital capacity only changes little with training, although water polo players exhibit higher amounts of air expelled after maximal inspiration than e.g., basketball, handball or soccer players (Durmic et al., 2015). Due to the innate character of this feature of the respiratory system, it might also be useful for the talent classification in the six sports investigated in this study.

Hemoglobin mass was also suggested for talent identification purposes (Eastwood et al., 2012) as it is not only relevant in endurance sports, but also could predict future success at least in young soccer players (Prommer et al., 2018).

### Statistical Analysis

All data were analyzed with SPSS (Version 25.0; SPSS Inc., Chicago, IL, United States) and statistical significance was set at *p* < 0.05. All test data were collected from the ninth grade classes on September 30th, 2016. The discriminative validity of the eighteen morphological, two motor, and three physiological measures was determined using a classification of athletes by means of a linear discriminant analysis (DA) and a nonlinear neural network MLP. In both classification procedures, the six sports served as the dependent grouping variable, whereas the test results were used as an independent variable set. The stepwise DA was based on the “leave-one-out” method. This means the classification of each individual was calculated using a function derived from all other cases without the single one case that was held out for final classification. Similarly, for the MLP analysis, three subsets were created for (i) training, (ii) testing of the predictive model, and (iii) the final classification of the left-out cases. Subsequently, the MLP was trained with 80% of all cases, whereas ten percent was used for testing the trained network. Finally, the classification was calculated for the hold-out of the remaining ten percent of cases. This specific type of leave-out strategy was repeated ten times so that each case should at least once belong to the left-out athletes that were finally classified. To quantify the validity of this talent identification strategy, the percentage of correct hits of the neural network classification was averaged over the ten trials and the mean value was used from there on. The classification quality of both methods was expressed by the proportion of correct hits, and was also classified as the percentage of athletes that were assigned as true positives to their own sport. An athlete was defined as false positive if he was classified as a participant of a specific sport for which he did not practice.

## Results

### Classification by Linear Discriminant Analysis and Nonlinear Neural Network

In the DA, three cases of the fencers were sorted out due to missing data. In a first attempt, a DA with the remaining total sample of *n* = 94 cases was calculated and a classification rate of 98.9% was obtained. In this analysis, only one table tennis player was assigned erroneously to judo. In a second attempt, a cross-validated DA was applied, where each of 94 athletes was iteratively used as a single hold-out case which has to be solely classified. On the basis of this leave-one-out procedure, 71.3% of all athletes were classified correctly and assigned as true positives to their own sport (Table 1). The best classification result of 85.0% correct hits was obtained in fencing, where only three out of twenty athletes were collated as false negatives to another sport (two athletes to volleyball, one athlete to table tennis). The highest fraction of false negatives was found in basketball (28.6%), mostly due to an erroneous assignment of four (57.1%) youth basketballers to the fencing group, and one (14.3%) to the volleyball group.

**Table 1:** Original and cross-validated classification of *n* = 94 single cases of youth athletes from six different sports on the basis of the 25 performance characteristics.

			Fencing	Basketball	Volleyball	Table tennis	Judo	Swimming	Total
Cross-validated	N	Fencing	17	0	2	1	0	0	20
		Basketball	4	2	1	0	0	0	7
		Volleyball	2	1	16	0	1	2	22
		Table tennis	2	0	1	11	1	0	15
		Judo	0	0	0	2	14	4	20
		Swimming	0	0	1	0	2	7	10
	%	Fencing	85,0	, 0	10,0	5,0	, 0	, 0	100,0
		Basketball	57,1	28,6	14,3	, 0	, 0	, 0	100,0
		Volleyball	9,1	4,5	72,7	, 0	4,5	9,1	100,0
		Table tennis	13,3	, 0	6,7	73,3	6,7	, 0	100,0
		Judo	, 0	, 0	, 0	10,0	70,0	20,0	100,0
		Swimming	, 0	, 0	10,0	, 0	20,0	70,0	100,0

**FIGURE 1:**
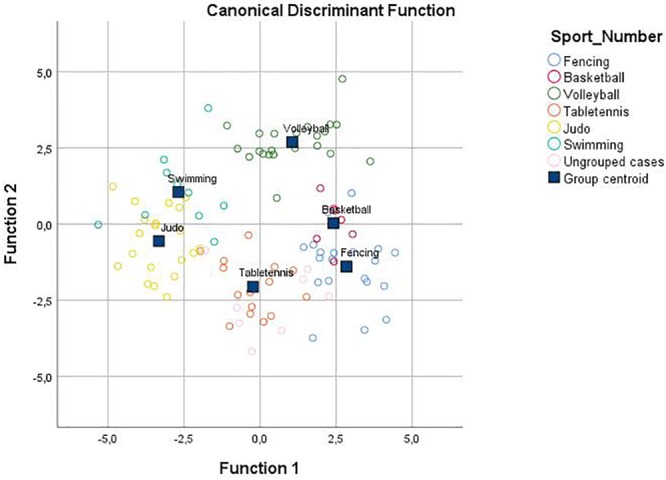
Plot of the individual and group differences between the six sports resulting from eighteen morphological, two motor, and five physiological tests. Functions at group centroids: Fencing, function 1 = 2.84 and function 2 = –1.40; Basketball, function 1 = 2.41 and function 2 = 0.03; Volleyball, function 1 = 1.06 and function 2 = 2.70; Table tennis, function 1 = –0.24 and function 2 = –2.06; Judo, function 1 = –3.33 and function 2 = –0.56; Swimming, function 1 = –2.68 and function 2 = 1.05.

Since there were six different sport groups, six linear discriminant functions were established. The first two functions accounted for 77.5% of the variance and are represented in Figure 1 on the *X*- and *Y-axes*. The athletes from the six sport groups are distributed around their respective centroids, which are located on distinct areas of the plot. The first function (Eigenvalue: 5.92) was the most important, accounting for 50.4% of the variance and was related primarily to morphological body dimensions. The second function (Eigenvalue: 3.18) accounted for 27.1% of the variance.

In the neural network analysis, the same three cases of fencers with incomplete data were cut out. In accordance with the linear analysis described above, in a first attempt, the MLP was trained with all complete cases (*n* = 94). According to the learning character of neural networks methods, the MLP also makes minimal assumptions in regard to relations within the data. Thus, the MLP is able to determine linear as well as nonlinear relationships by its iterative learning mechanism. Along the learning process of neural networks, the prediction result may partly depend on the start vector that is set in a random mode; consequently, the results of the MLP may vary slightly from repetition to repetition (Pion et al., 2016). Therefore, all MLP analyses were repeated ten times to secure that the mean value of this series represents the overall quality of the results obtained by means of that neural network method.

The application of the nonlinear MLP in a first attempt was based on the total sample of *n* = 94 athletes, and the ten trials led to an average classification rate of *M* = 99.3%, indicating that on average 93 of 94 original cases were collated correctly in regard to their respective sport. In the row of the ten repetitions of the MLP analysis, on only three occasions were one, two, and three athletes assigned to any other sport; in the other seven repetitions, 100% correct hits were calculated. In a second attempt, a cross-validated MLP analysis was applied, where in each of the ten repetitions 80% of the 94 athletes were used as training data set to calibrate the network, ten percent of the cases were taken to test the network solution, and the remaining ten percent of cases served as a hold-out data set for the cross-validated prediction of the sport group in which these athletes performed. On the basis of this leave-ten-percent-out procedure, 71.0% of the 94 athletes were classified correctly and assigned as true positives to their own sport (Table 2). The best classification result of 83.4% correct hits were obtained in volleyball, where only an average four of 22 athletes were collated as false negatives to another sport (leaning toward judo and swimming). The smallest fraction of true positives was found in basketball (20.0%), mostly due to an erroneous assignment of the MLP of most youth basketballers to the fencing group and somewhat fewer to the volleyball group.

**Table 2:** Cross-validated classification results (mean value from ten repetitive calculations) from the nonlinear neural network analysis (multilayer perceptron).

	Basketball (*M*_percent_)	Fencing (*M*_percent_)	Judo (*M*_percent_)	Swimming (*M*_percent_)	Table tennis (*M*_percent_)	Volleyball (*M*_percent_)
Basketball (*n* = 7)	20.0					
Fencing (*n* = 23)		70.7				
Judo (*n* = 20)			80.0			
Swimming (*n* = 10)				37.5		
Table tennis (*n* = 15)					83.3	
Volleyball (*n* = 22)						83.4

### Prioritization of Talent Characteristics for Each Sport

To analyze which particular talent features specifically distinguish between the participants of one specific sport and all other athletes, three stepwise DA and MLP analyses were calculated. The aim was to prioritize the most relevant talent characteristics of the respective single sport. In both analyses, only two groups were formed and served as the dichotomic dependent variable: one group of participants of the single sports discipline under investigation, and a second group of all other athletes from the remaining five sports. Descriptive statistics of the 25 variables measured in the *N* = 97 male athletes, which were included into the three stepwise DA and MLP analyses as documented in Table 3. Also, the mean values of the test age are reported, which do not differ systematically between the six sport groups (*F*_5;91_ = 1,90; *p* = 0.102).

**Table 3:** Morphological, motor, and physiological prerequisites of the athletes from six different sports.

	Basketball (*n* = 7)	Fencing (*n* = 20)	Judo (*n* = 20)	Swimming (*n* = 10)	Table tennis (*n* = 15)	Volleyball (*n* = 22)
Age (mon)	179.7 ± 6.7	179.1 ± 6.4	180.0 ± 6.6	172.8 ± 6.5	178.8 ± 7.5	177.0 ± 6.6
**Morphological characteristics**					
Body height (cm)	182.6 ± 5.5	178.9 ± 7.3	* 177.7 ± 7.3	180.0 ± 7.1	170.7 ± 4.0	* 192.4 ± 3.3
Sitting height (cm)	94.1 ± 2.4	* 94.5 ± 3.9	93.6 ± 3.4	94.3 ± 3.6	90.4 ± 3.6	100.3 ± 2.8
Arm span (cm)	182.9 ± 6.8	179.7 ± 7.3	181.5 ± 7.6	184.3 ± 7.2	173.3 ± 3.9	195.7 ± 4.6
Arm length (cm)	78.1 ± 3.9	77.0 ± 3.4	77.6 ± 3.1	79.2 ± 3.9	74.5 ± 1.5	83.5 ± 2.1
Leg length (cm)	96.4 ± 3.9	93.0 ± 4.0	* 92.6 ± 4.7	94.9 ± 6.3	88.3 ± 3.0	100.7 ± 2.5
Low leg length (cm)	* 49.6 ± 2.7	47.6 ± 2.6	* 47.7 ± 2.6	47.7 ± 2.1	§* 44.3 ± 1.0	51.4 ± 1.1
Shoulder width (cm)	* 37.7 ± 1.5	§* 38.4 ± 1.5	40.3 ± 1.2	40.1 ± 1.8	38.5 ± 2.4	§* 42.6 ± 1.3
Crista width (cm)	§28.2 ± 0.9	28.2 ± 1.7	28.0 ± 1.6	* 27.0 ± 1.5	26.7 ± 0.8	* 30.3 ± 1.6
Chest girth (cm)	81.6 ± 4.6	§* 80.5 ± 4.3	* 92.8 ± 6.4	§* 90.3 ± 4.3	83.9 ± 5.4	91.0 ± 6.0
Calf girth (cm)	34.3 ± 0.9	36.5 ± 2.0	36.9 ± 2.5	* 36.2 ± 1.7	35.0 ± 2.8	38.9 ± 3.5
Waist girth (cm)	67.6 ± 1.5	69.5 ± 3.2	78.2 ± 7.5	74.2 ± 5.9	72.1 ± 3.3	79.7 ± 7.3
Thigh circumference (cm)	* 49.2 ± 1.4	* 52.6 ± 3.3	56.7 ± 5.1	§* 51.7 ± 3.3	51.8 ± 5.2	58.3 ± 4.6
Ankle circumference (cm)	21.7 ± 1.2	22.4 ± 1.2	* 22.7 ± 1.7	21.9 ± 1.1	21.1 ± 1.2	§23.6 ± 1.8
Achilles tendon length (cm)	25.8 ± 2.1	* 23.6 ± 1.4	23.0 ± 2.4	* 25.4 ± 1.3	21.9 ± 2.9	* 26.3 ± 1.2
Subscapular angle (deg)	6.4 ± 1.4	7.4 ± 1.2	§* 11.4 ± 3.5	8.9 ± 3.0	6.9 ± 1.4	* 9.7 ± 3.6
Abdomen skinfold thickness (mm)	7.7 ± 1.2	8.4 ± 2.4	15.2 ± 9.3	9.4 ± 4.5	9.7 ± 5.8	12.8 ± 7.3
Upper arm skinfold thickness (cm)	8.9 ± 0.9	9.3 ± 2.9	12.3 ± 4.6	8.6 ± 2.7	8.7 ± 3.7	11.0 ± 4.9
Body weight (kg)	59.4 ± 2.7	63.6 ± 7.6	73.4 ± 11.0	66.8 ± 9.5	60.5 ± 8.3	82.1 ± 10.5
**Motor characteristics**					
Dynnamic back strength (kg)	§* 88.7 ± 15.5	§* 96.7 ± 15.3	§* 123.3 ± 17.0	102.7 12.6	§* 94.8 ± 16.5	* 114.7 ± 18.5
Simple-reaction time (ms)	217 ± 33	218 ± 24	236 ± 27	213 ± 29	233 ± 35	§230 ± 24
**Physiological characteristics**					
Resting heart rate (bpm)	67.7 ± 6.2	62.7 ± 6.2	* 60.7 ± 7.3	65.1 ± 5.9	67.5 ± 4.7	65.0 ± 6.4
Vital capacity (ml)	4121 ± 295	§4290 ± 579	4420 ± 793	§* 5071 ± 863	§* 3823 ± 462	§* 5067 ± 1114
Hemoglobin mass (mg)	130.6 ± 12.2	140,1 ± 8.4	§* 144.3 ± 7.1	138.5 ± 8.7	136.2 ± 11.0	§*128.5 ± 11.2
Blood pressure (systolic; mmHg)	115.3 ± 9.3	120.2 ± 10.3	117.2 ± 13.2	122.7 ± 7.3	119.3 ± 10.7	122.4 ± 11.7
Blood pressure (diastolic; mmHg)	66.4 ± 8.4	69.7 ± 6.8	* 65.0 ± 8.4	§*63.0 ± 11.6	* 72.5 ± 7.7	70.0 ± 8.2

*^∗^Significant *F-value* in the stepwise DA; §Characteristics with an average of more than 80% normalized importance in the ten MLP repetitions.*

#### Basketball

In the stepwise DA of the anthropometrical measures, it was shown that the youth basketball players differed from the rest of the sport groups in lower leg length (*F* = 15.41 and *p* < 0.05), shoulder width (*F* = 7.78 and *p* < 0.05), and thigh circumference (*F* = 3.41 and *p* < 0.05). In contrast, the MLP identified the crista width (92.6% importance) wider in these individuals than in athletes from other sports. For motor characteristics, the DA, as well as the MLP, confirmed that dynamic back strength (*F* = 5.83 and *p* < 0.05, and 90.4% resp.) was lower in the basketballers than in the other athletes. In regard to the physiological features, no parameter exhibited a higher standardized importance than 80%.

#### Fencing

In the DA, five anthropometrical measures of fencers differed significantly from the total group of all other youth athletes from the remaining five sports. Sitting height (*F* = 20.45 and *p* < 0.05), shoulder width (*F* = 23.52 and *p* < 0.05), chest circumference (*F* = 38.31 and *p* < 0.05), thigh circumference (*F* = 13.79 and *p* < 0.05), and also Achilles tendon length were smaller in comparison to those of their counterparts belonging to other sports. The MLP analysis confirmed by a standardized importance of more than 80% that shoulder width (85.0%) and especially chest circumference (97.4%) in fencers are less developed than in athletes belonging to the other five sport disciplines. For motor characteristics, the DA, as well as the MLP, confirmed that dynamic back strength (*F* = 6.50 and *p* < 0.05, and resp. 89.9%) is lower than in athletes from other sports. In the row of the physiological variables, the vital capacity (92.2% importance) was identified by the MLP as being less voluminous than vital capacity for other sports.

#### Judo

In judo, the stepwise DA led to a comparably long list of significant anthropometrical variables that discriminated between judo athletes and the other youth athletes. Body height (*F* = 14.45 and *p* < 0.05), leg length (*F* = 15.84 and *p* < 0.05), and lower leg length (*F* = 25.03 and *p* < 0.05) were smaller than in athletes belonging to other sports. On the other hand, chest circumference (*F* = 16.46 and *p* < 0.05), ankle circumference (*F* = 6.16 and *p* < 0.05), and also subscapular angle (*F* = 4.78 and *p* < 0.05) turned out to be greater than those of their counterparts from other sports. The relevance of the subscapular angle is especially corroborated by the MLP analysis, which calculated a standardized importance of 89.7% for this body characteristic. In regard to the motor tests, the DA and MLP agreed that dynamic back strength distinguishes the most (99.5%) between judo athletes and the rest of the investigated total sample. For physiological measures, the stepwise DA identified a lower heart rate at rest, lower diastolic blood pressure, and higher hemoglobin mass as significantly different from the other sports. The high importance of hemoglobin mass (93.5%) was also confirmed by the nonlinear neural network method.

#### Swimming

In swimming, the stepwise DA identified five anthropometrical features that distinguished between swimmers and all other youth athletes. On one hand, the crista width (*F* = 4.82 and *p* < 0.05) and thigh circumference (*F* = 20.62 and *p* < 0.05) were smaller, and on the other hand, the chest and calf circumference (*F* = 28.81 and *p* < 0.05; *F* = 8.51 and *p* < 0.05), as well as the Achilles tendon length (*F* = 6.42 and *p* < 0.05), were greater than in the athletes from other sports. The importance of the positive and negative differences in the chest (97.5%) and thigh circumference (90.0%) were underlined by the neural MLP analysis. Both motor characteristics did not show differences between the swimmers and the U15/U16 athletes from the other five sports. In the set of physiological parameters, the stepwise DA as well as the MLP both stressed the significant relevance of the higher vital capacity (*F* = 6.41 and *p* < 0.05, and 96.3%) and the lower diastolic blood pressure (*F* = 5.15 and *p* < 0.05, and 84.9%) in swimmers.

#### Table Tennis

The stepwise DA in the table tennis players was in line with the result of the MLP as both methods identified shorter lower leg length (*F* = 36.74 and *p* < 0.05, and 88.0% resp.) as the only one anthropometric variable that was significantly different from the body dimensions of other youth athletes. For motor characteristics, DA and MLP confirmed that dynamic back strength (*F* = 5.62 and *p* < 0.05, and 94.8% resp.) in table tennis players is greater than in the athletes from other sports. Another noteworthy factor in the physiological variables is that both methods agreed that vital capacity (*F* = 11.41 and *p* < 0.05, and 96.2% resp.) is smaller in table tennis athletes, whereas only the stepwise DA discovered that diastolic blood pressure was lower in these players.

#### Volleyball

For the list of anthropometrical measures, the stepwise DA showed that youth volleyball players displayed greater body dimensions compared to the rest of the participants in regard to body height (*F* = 2.22 and *p* < 0.05), shoulder width (*F* = 13.86 and *p* < 0.05), crista width (*F* = 6.89 and *p* < 0.05), Achilles tendon length (*F* = 5.27 and *p* < 0.05), and subscapular angle (*F* = 3.93 and *p* < 0.05). The MLP confirmed greater shoulder width (83.9% importance) and reported that ankle circumference (84.4%) was also larger in the volleyballers. In regard to the motor diagnostics, the stepwise DA stressed significantly higher dynamic back strength (*F* = 6.19 and *p* < 0.05) in youth volleyballers, whereas the MLP stressed that youth volleyballers performed more slowly in the simple eye-hand-reaction test (84.9% standardized importance). Both linear and nonlinear methods furthermore revealed that in the set of physiological variables, vital capacity (*F* = 16.94 and *p* < 0.05, and 83.4% resp.) as well as hemoglobin mass (*F* = 22.14 and *p* < 0.05, and 88.0% resp.) were higher in the volleyballers compared to the remaining total group of elite youth athletes belonging to other sports.

## Discussion

The main objective of this research was to discriminate between youth athletes from an elite sport school—many of whom will contribute to the next generation of elite senior athletes—belonging to the six researched sports in the Shanghai province. Therefore, a generic test battery of 25 physiological, anthropometric, and motor characteristics was administered to 97 young elite athletes. It should be highlighted that, in this study, linear and nonlinear statistical methods were parallelly used to identify the most relevant talent characteristics of each of the six sports and reversely confirm the results of each method. The main findings included the high discriminative validity of the generic test battery that allowed the original correct assignment of 98.9% of cases by means of the discriminant analysis. The quota of 98.9% correct assignments in the total group of 94 youth athletes is in line with the 96.4% reported by Pion et al. (2015) for a selection of nine different sports (badminton, basketball, gymnastics, handball, judo, soccer, table tennis, triathlon, and volleyball), and the 100% reached by Pion et al. (2014) in a classification analysis in the three martial arts, judo, karate, and taekwondo. Especially, when compared to the 88.0% classification rate of Leone et al. (2002) in a group of figure skaters, swimmers, tennis and volleyball players, our results seem to be quite high. Also Opstoel et al. (2015) reported a lower figure of 85.2% for a discriminant analysis in ball sport, dance, gymnastic, martial arts, raquet sports, and swimming athletes. Our result of 71.3% obtained from the discriminant analysis by means of the leave-one-out validation strategy cannot be compared directly to the findings of the research groups mentioned above, as these researchers calculated the predictive accuracy only for the original sample including all members of the total groups. When compared to the 76.8% correct DA predictions for the left out participants reported in the study of Hohmann et al. (2015), our results are still satisfactory. The best classification results of 83.4% correct hits were obtained in volleyball, which is in line with Leone et al. (2002), who attributed this finding to the relevance of the anthropometric make-up in this particular sport. In the neural network analysis by means of the MLP tool and the use of the ten-percent-hold-out strategy, we discovered that 71.0% of classified correctly into their original sport. Although, that we expected that the nonlinear neural network method would allow for a higher classification rate than the linear discriminant analysis, the equality with the DA result is in line with the findings of Hohmann et al. (2015), who reported 69.6% correct predictions by means of the MLP analysis in German youth athletes of the same age group.

The comparison of our results of the linear and nonlinear classification of the Chinese youth elite athletes with the results from the aforementioned European studies shows that the athletic make-up of the participants from the Shanghai sport school does not seem to differ systematically from that of their European counterparts. This finding might be related to the homogenization effect of the sports-specific demands posed by the international competition system.

Furthermore, in our study there were still deficits in prediction accuracy, as the origin of about one third of the participants in regard to the six different sports could not be identified correctly. This finding might be attributed to the selection of the tests. In the past, talent identification for certain sports was based primarily on sport-specific testing, which made it difficult to compare results between different sports. Also, such sport-specific tests pose the problem that athletes from one sport who are not familiar with the specific techniques and skills of an alternate sport cannot perform tests other than those specific to their own sport with a reliable and valid personal outcome. Therefore, it is paramount that talent identification programs consist of a multidisciplinary mixture of anthropometric, motor, psychological, or physiological testing methods of low specificity to allow for a more complete assessment of each athlete as well as between-sports comparisons (Pion et al., 2014, 2015, 2016). With this in mind, the results of this study provide substantial information about the validity of the anthropometric, motor, and physiological tests for the discrimination of young athletes from different sports; furthermore, we are provided with evidence for the applicability of the different measures for talent identification in the six sports investigated.

The combination of linear DA and artificial neural networks is a fruitful approach for resolving the problem of talent identification specifically when it is assumed that different types of talent patterns exist in the make-up of promising youngsters, which may lead to the same performance outcome in particular elite sports (Philippaerts et al., 2008; Pfeiffer and Hohmann, 2012; Pion et al., 2016; Till et al., 2016). As both methods on the basis of the original data set led to a quite similar and almost perfect quota of 98.9 (DA) and 99.3% (MLP) correct assignments to the six sport disciplines, both classification methods seem to establish an almost linear relationship between the 25 predictors and the six categories of sports. This assumption is corroborated by the results of the cross-validated attempts which also showed an almost identical overall quality of 71.3% (DA) and 71.0% (MLP) correct hits in the 94 individual cases. The only relevant difference between the results of the two analytical models was found in regard to the sport with the highest fraction of correct predictions. Whereas the DA identified 85.0% of the fencers correctly, the MLP was most precise in the volleyball group (83.4%). On the other hand, the very similar and rather poor prognostic results of both methods in the group of basketballers (28.6% for DA, 20.0% for MLP) may partly depend on the small sample size of only seven players; although, both predictions were still higher than the random quota (16.7%).

The results in regard to the importance of the single talent characteristics obtained in each particular sport are in agreement with previous research conducted on performance profiles in these respective sports.

### Physiological Characteristics

It is well-known that the heart rate at rest along with systolic and diastolic blood pressure are lower for endurance sports (Cornelissen et al., 2010). Moreover, within those sport categories, the reduction of blood pressure is more pronounced in disciplines with higher exercise intensities (Goldring et al., 2014). Thus, it is not surprising that, in our study, both the resting heart rate and resting diastolic blood pressure of judo participants contributed significantly to the discrimination of swimmers and judo athletes from those belonging to other sports.

In swimming and volleyball, vital capacity of youth athletes also turned out to be higher, which helped distinguish these players from players belonging to other sports; fencing and table tennis groups could especially be identified by their lower values in this category.

The significant contribution of a higher hemoglobin mass to the classification of judo athletes and the discrimination of this group from all other study participants is somewhat unexpected because former research by Malczewska-Lenczowska et al. (2013) found that, when compared to endurance athletes, even elite judo athletes exhibit significantly lower values of hemoglobin mass related to body weight.

### Anthropometric Characteristics

The basic anthropometric features (body height and particular body segment lengths) have been proven relevant for youth expert athletes in basketball (Ziv and Lidor, 2009), swimming (Hohmann et al., 2018), and volleyball (Lidor et al., 2007).

The Achilles tendon length is positively related to the running economy (Hunter et al., 2011, 2015) and is also relevant for jumping power (Earp et al., 2011). Thus, it is not surprising that youth elite swimmers and volleyballers possessed longer tendons since in both sports a powerful jump-off from the start block (Rebutini et al., 2016; Amaro et al., 2017) and floor (Sattler et al., 2012; Battaglia et al., 2014), respectively, is a regular necessary action; additionally, a highly-exerted push-off against the wall in swimming contributes significantly to a swimmer’s overall performance. Interestingly, swimmers and fencers exhibited a smaller thigh circumference, which helped discriminate them significantly from the total group of other athletes; this particular characteristic may be attributable to the reduced body weight of the participants belonging to both sports.

The inferior subscapular angle is influenced by the muscle volume of the upper body because it is attached to the m. teres major and covers the m. latissimus dorsi. In our study, this feature was larger in judo and volleyball athletes. In Judo, this result matches the findings of Drid et al. (2015), who pointed out that the subscapular angle is greater in elite judo competitors at the international level compared to sub-elites performing only at the national level.

### Motor Characteristics

Greater upper body muscle volume, which in both judo and volleyball corresponds with better performances in the back strength test, contributed to their differentiation from other sports. Greater strength prerequisites in judo athletes that support throwing, sweeping, and clamping actions during a fight were already reported by Franchini et al. (2011). These studies agree with our discovery that higher dynamic back muscle strength of the lumbar spine contributed significantly to the discrimination of judo and volleyball athletes from the other sport disciplines. In judo, this finding corroborates the early research of Kort and Hendriks (1992), who attributed this advantage to a more specific training regimen in elite judo cadres. In elite volleyball, back squat exercises contribute an important portion of the daily training routine with the aim of creating a balanced trunk musculature and core stability, thus protecting tall volleyballers from the elevated risk of lower back pain (Ezechieli et al., 2013).

Research on single reaction time in sports is abundant, but due to different tasks, test protocols, and performance levels, the findings are hardly comparable. As a general result, one can confirm the assumption that elite athletes from most sport disciplines differ from non-athletes when performing generic, single, and elementary reaction tasks. This holds true in basketball (Bhabhor et al., 2013), judo (Badau et al., 2018), table tennis (Nakamoto and Mori, 2008), and volleyball (Kokubu et al., 2006), but maybe not in fencing—at least when compared to fencing novices as opposed to non-athletes from the normal population (Harmenberg et al., 1991). In elite swimmers, to our knowledge, generic single reaction time has not yet been compared to the untrained population. In conclusion, the finding of this study, which concludes longer single task reaction times significantly distinguished volleyballers from the other athletes, cannot be derived sufficiently from the existing literature and therefore needs further investigation.

Our study has several limitations. The first limitation is the relatively small sample size, especially in basketball (*n* = 7) and swimming (*n* = 10), which was a consequence of the superior quality of athletes in a Chinese elite sport school. All applicants undergo a strict sport-specific selection procedure at an early age, which implies that members of the investigated age group of 14–15 year-old male athletes cannot be numerous by definition. A second limitation is the focus solely on male youth athletes. Besides the even smaller numbers of female youth athletes in the Shanghai sports school, the focus on solely male athletes also resulted from the complexity of the study due to the different influence of the gender-specific athletic make-up on the sports-specific performances of male and female youth athletes in most of the sports disciplines under investigation.

## Conclusion

The results of this study reveal that in Under-15 and Under-16 male athletes from a Chinese elite sport school in Shanghai, the between-sports differences in a battery of generic anthropometric, motor, and physiological tests allow one to distinguish more than two out of three young athletes’ talents according to their individual sport provenience, independent from the classification method (DA: 71.3%; MLP: 71.0%) used. Furthermore, the overall accuracy of the talent classification in the Chinese elite youth athletes corresponds to the level found in European studies. To allow for such kind of between-sports comparisons, it is necessary that talent identification programs consist of a multidisciplinary mixture of anthropometric, motor, psychological, or physiological testing methods of low specificity. The linear and nonlinear statistical methods that were used in parallel to identify the most relevant talent characteristics of each of the six sports by means of the leave-one-out procedure reversely confirmed the quality of the results. In regard to the relevance of the different sports-specific talent characteristics for talent identification campaigns in the practical fields of the six sports, the applied talent classification strategies underlined the importance of superior stature measures solely in volleyball. Besides longer Achilles tendons in swimmers and volleyball players, a more pronounced chest circumference was found in swimmers and judo athletes. In regard to the motor characteristics, a high back strength turned out to be helpful to discriminate between the six sports. Notably in judo, table tennis, and volleyball players this athletic performance prerequisite was specifically important. In the series of the physiological characteristics, vital capacity was relevant in swimming and volleyball, and hemoglobin mass was elevated in judo and volleyball athletes. A lower heart rate at rest was detected in judo participants alone.

The limitation of the relatively small sample size and the focus on solely male athletes needs further investigations in the talent make-up of elite youth sport cadres. Furthermore, especially a greater variety of motor tests including also speed, endurance and flexibility tests in the youth athletes assessments were reasonable.

## Ethics Statement

This study was carried out in accordance with the recommendations of “Science Research Ethics Committee at the Shanghai University of Sport” with written informed consent from all subjects. All subjects gave written informed consent in accordance with the Declaration of Helsinki. The protocol was approved by the “Science Research Ethics Committee at the Shanghai University of Sport.”

## Author Contributions

KZ was in charge of data collection in elite sports school. AH was in charge of data analysis. YC and BZ were in charge of testing in elite sports school. JP was in charge of revising the manuscript. BG was in charge of financial support and test organization.

## Conflict of Interest Statement

The authors declare that the research was conducted in the absence of any commercial or financial relationships that could be construed as a potential conflict of interest.
